# Epileptic Seizures After Allogeneic Hematopoietic Stem Cell Transplantation

**DOI:** 10.3389/fneur.2021.675756

**Published:** 2021-07-16

**Authors:** Zhuo Wang, Munan Zhao, Sujun Gao

**Affiliations:** ^1^Department of Hematology, The First Hospital of Jilin University, Changchun, China; ^2^Department of Oncology, The First Hospital of Jilin University, Changchun, China

**Keywords:** allogeneic hematopoietic stem cell transplantation, complications, epileptic seizures, neurotoxicity, status epilepticus

## Abstract

Technique in allogeneic hematopoietic stem cell transplantation has greatly advanced over the past decades, which has led to an increase in the number of patients receiving transplantation, but the complex procedure places these transplant recipients at high risk of a large spectrum of complications including neurologic involvement. As a common manifestation of neurological disorders, epileptic seizures after transplantation have been of great concern to clinicians because it seriously affects the survival rate and living quality of those recipients. The aim of this review is to elucidate the incidence of seizures after allogeneic hematopoietic stem cell transplantation, and to further summarize in detail its etiologies, possible mechanisms, clinical manifestations, therapeutic schedule, and prognosis, hoping to improve doctors' understandings of concurrent seizures following transplantation, so they can prevent, process, and eventually improve the survival and outlook for patients in a timely manner and correctly.

## Introduction

**Figure 1:**
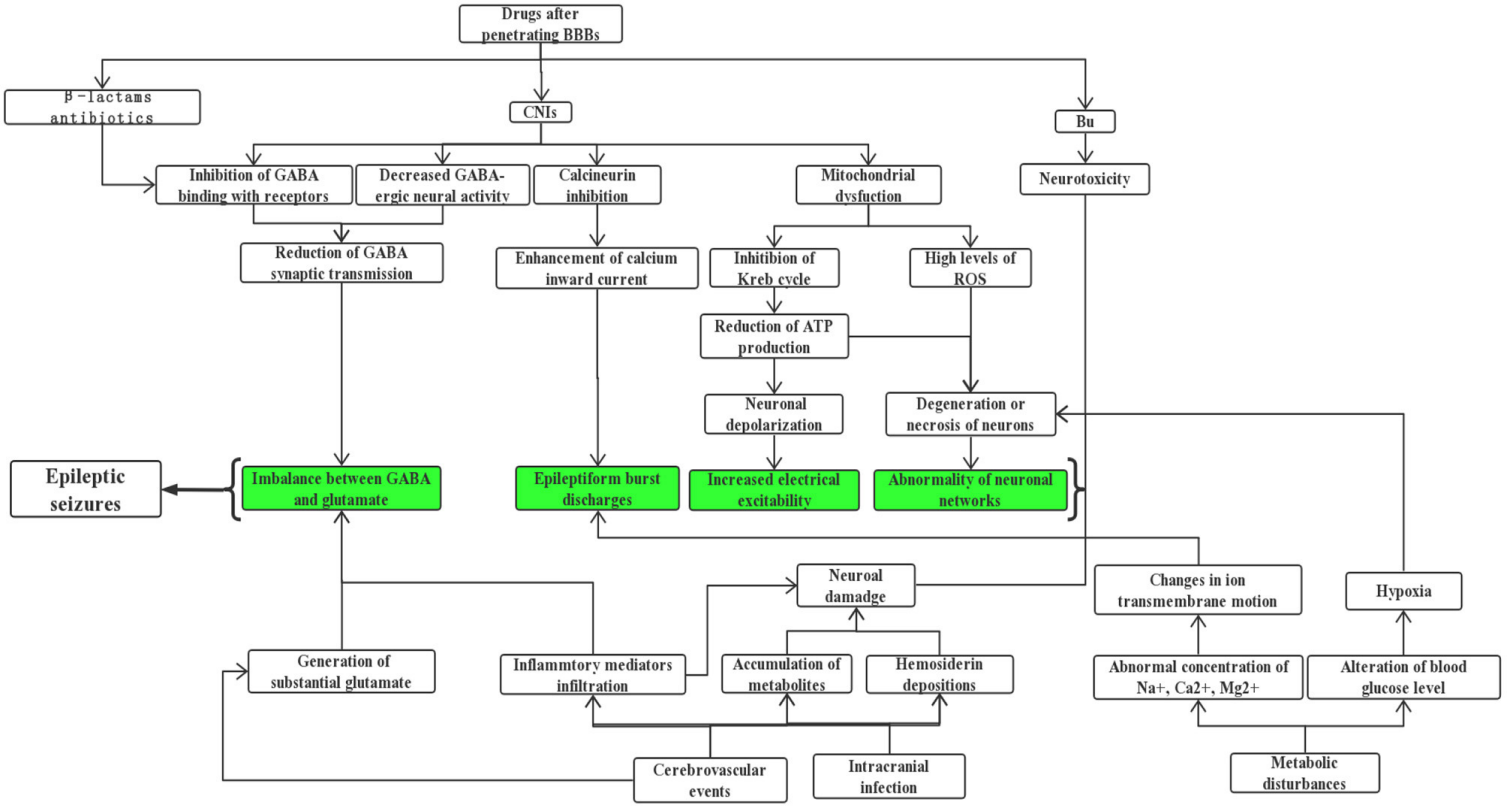
The main mechanisms of seizures after allo-HSCT.

Allogeneic hematopoietic stem cell transplantation (allo-HSCT) has been considered the only curative treatment for some malignant hematological diseases, and the long-term survival rate of patients with these hemopathies improved dramatically because of the continuous improvements and maturation in bone marrow transplant procedures and supportive care (1). But as a high-risk therapy, HSCT is still associated with a series of complications including the involvement of the central nervous system (CNS). Studies have reported an incidence of neurologic complications after HSCT ranging from 6.6 to 70% (2–8), such a wide span may be explained by the heterogeneity of study populations and extensive differences in definitions of CNS complications. An epileptic seizure is one of the most common symptoms of neurological disorders, they can manifest as strange sensations, emotions, and behaviors, or sometimes convulsions, muscle spasms, and loss of consciousness, eventually leading to serious impairments of normal CNS function and even death. Several clinical studies have indicated that seizures are a relatively uncommon but life-threatening symptom of neurologic complication after allo-HSCT, with incidence ranging from 1.6 to 15.4% (4, 9–12), and those transplanted recipients with seizures often have poor prognosis and lower quality of life, so they should be paid more attention to by clinicians. Unfortunately, there is limited literature focusing on this subject and unified therapeutic approaches are still lacking. Herein, we aim to provide a comprehensive summary about the etiologies (Table 1) and possible underlying mechanisms (Figure 1) of seizures after allo-HSCT, its clinical features, treatment, and prognosis are also described; a better understanding of these basic aspects is of paramount importance to help doctors prevent related risk factors earlier, treat properly and promptly, and finally improve patients' clinical outcomes.

**Table 1:** Etiologies of seizures following allo-HSCT.

**Categories**
**Drug-related neurotoxicity**
Chemotherapy (Bu)
Immunosuppressants (CsA, TCA)
Other drugs (antibiotics: unsubstituted penicillin, fourth-generation cephalosporins: imipenem, fluoroquinolones: antifungal - azole, antiviral agents - foscarnet)
**Metabolic disturbances**
Hyponatremia, hypomagnesemia, hypocalcemia, hypernatremia, hypoglycemia
Blood glucose abnormalities
End-stage organ failure (hepatic or uremic encephalopathy)
**Intracranial infection**
Viruses (human herpesvirus-6, cytomegalovirus, herpes simplex virus)
Fungi (Aspergillus, Candida)
Bacteria (G+, G–)
**Cerebrovascular events**
Hemorrhagic diseases (intraparenchymal hemorrhage, subdural hematoma, subarachnoid, hemorrhage, multiple hemorrhage lesions)
Cerebral thrombotic events
Transplantation-associated thrombotic microangiopathy
**Others**
Post-transplantation lymphoproliferative disorders (PTLD)
Relapse of CNS
Secondary neoplasm (meningioma, glioblastoma, astrocytoma, etc.)
Vasculitis or immune-mediated encephalitis caused by cGVHD
Unknown

## Etiologies and Possible Mechanisms of Post-Transplant Seizures

### Drug-Related Neurotoxicity

#### Chemotherapy

During the implementation of allo-HSCT, drug-related adverse effects have always been a clinical concern, especially when a convulsive seizure occurs in the early phase of transplantation; medication factor is theoretically the primary consideration. It is known that busulfan (Bu) is frequently employed in conditioning regimens for patients undergoing HSCT, high-dose Bu can freely penetrate the blood-brain barrier (BBB), leading to an almost similar level of Bu concentration in both CNS and plasma (13), and it performs its neurotoxic effects through damaging neurons, so adjustment of administration dosage by monitoring Bu concentration is recommended in clinic (14). Previous studies have reported a seizure rate of ~10% (range 1.8–40%) after Bu usage in adults when there were no prophylactic anticonvulsants (15, 16), furthermore, up to 60% of patients may present with an abnormal electroencephalogram (EEG) even without significant seizure symptoms (17, 18). This phenomenon further confirms its neurotoxic effects. Under this circumstance, phenytoin sodium has been routinely utilized to prevent busulfan-induced seizures (BIS) in clinical practice for years though there still exists a frequency of 0–5.5% in the incidence of seizures (16, 19, 20). Given the facts that phenytoin sodium has the disadvantages of being a strong inducer of cytochrome P450 (CYP) hepatic enzyme, which influences the metabolism of cyclophosphamide in the conditioning schedule of HSCT recipients, together with its narrow therapeutic window and the advent of new antileptic drugs, new research efforts in some SCT centers have shifted to using alternatives as prophylaxis against BIS, such as benzodiazepines, valproate, etc. (16, 21–24). Levetiracetam stands out because it fulfills many of the properties of an ideal prophylactic agent, for example, it has ~100% bioavailability, it lacks a significant drug-drug interaction, and can be rapidly loaded to effective doses, what is more, it shares a similar outcome with phenytoin in seizure prevention and overall effects (19, 21, 22, 25). Chaguaceda et al. (26) have suggested that the optimal dose of oral levetiracetam to prevent BIS in adults seemed to be 1,000 mg every 12 h, starting 12 h before the administration of iv Bu until 48 h after the last dose. In the future, more prospective studies with larger populations are necessary to validate its efficacy, safety, and appropriate dosage to act as a first-line anticonvulsant for BIS prevention.

#### Immunosuppressants

Calcineurin inhibitors (CNIs) are a class of widely used drugs to prevent graft vs. host disease (GVHD) and its sequelae after HSCT. Cyclosporin A (CsA) and tacrolimus (TAC) are mainly included, they exert immunosuppressive actions by binding to immunophilins to inhibit the calcineurin-mediated calcium-dependent signaling pathways that mediate the formation of interleukin-2 and T-lymphocyte activation (27). Although many patients benefit from them, neurotoxicities caused by these agents have remained serious, a seizure is one of the severe manifestations which cannot be ignored. Based upon a retrospective study of over 1461 recipients of HSCT by Zhang et al. (10), more than 40% of seizures are associated with drug application, especially the usage of immunosuppressants which are listed at the top of reasons for epileptic seizures; this result is consistent with other similar investigations (12, 28). Nevertheless, the occurrence of seizures is not positively correlated with the blood concentration of CsA, this might be attributable to the inter-individual variation in the metabolism of CsA (29).

Previous reports have indicated that both CsA and TAC enhance the permeability of the blood-brain barrier (BBB) by damaging tight junctions directly, inducing apoptosis of brain capillary endothelial cells (30), and decreasing P-glycoprotein's efflux pump function (27, 31). In addition, CsA exposure can increase nitric oxide (NO) production in brain endothelial and astroglial cells, further mediating endothelial injury and impairment of BBB function (32, 33). But the underlying mechanism through which CNIs cause convulsions after penetrating the BBB has not yet been fully elucidated, although several explanations have been proposed: (1) There is evidence (34, 35) showing that CsA can decrease gamma aminobutyric acid (γ-GABA) neural activity. Meanwhile, it can inhibit GABA from binding to its receptors, eventually influencing synaptic transmission of this inhibitory neurotransmitter and breaking down the balance with those excitatory ones. (2) Klawitter et al. (36) drew the conclusion that CsA gave rise to mitochondrial disorder, mainly reflecting in the inhibition of the Krebs cycle and reduction of adenosine triphosphate (ATP). In this case, nerve cells are prone to depolarize and discharge, which lays the foundation of seizures. Besides, a derailment of mitochondrial oxidative phosphorylation probably results in the release of a high level of reactive oxygen species (ROS) (37, 38), which can induce loss of neural viability and a drop in the number of neurons; these changes are of great value in increasing susceptibility to seizures. (3) It is well-recognized that calcineurin is widely distributed in multiple sites of the brain, for instance, the cerebral cortex, striatum, cerebellum, substantia nigra, and hippocampus, where it regulates the activity of ion channels, synaptic plasticity, and even neurotransmitter release (27, 39). Inhibition of its critical roles by CNIs brings about ion channel dysfunction, especially the enhancement of calcium inward current, eliciting epileptiform burst discharges (40, 41). (4) The breakdown of the BBB can further lead to vasogenic edema, with a predilection for the posterior cerebral white matter, which matches the earliest pathogenesis of posterior reversible encephalopathy syndrome (PRES). This disease is characterized by a series of neurologic symptoms, ranging from headache, seizures, confusion, visual disturbances, and so on, of which seizures occur in more than 70% of patients who suffered from PRES (6, 31). From the perspective of clinical electrophysiology, Grioni et al. (42) even regarded it as a posterior unilateral reversible epileptic disorder in essence. If there is prolonged exposure to CNIs, cytotoxic edema resulting from mitochondrial dysfunction and reduced NO production exists simultaneously, this maybe an early indication of irreversible damage (31, 33, 43). Besides, other investigators believed that CNI-induced convulsions might be associated with gene polymorphism and other factors (29, 44–46). Interestingly, there are studies reporting that CNIs may behave as neuroprotective agents in animal models of epilepsy (47, 48), which contradicts the current understanding, so additional investigations are needed to clarify the key influential factors of determining whether it exerts a toxic or protective effect.

#### Other Drugs

Prophylactic or therapeutic usage of certain antibiotics in both pre and post transplantation periods can also trigger epileptic seizures or status epilepticus (SE) despite a relatively rare incidence. It has been documented in reviews (49–51) that a high dosage of β-lactams has epileptogenic potential, which is most commonly found in patients prescribed with unsubstituted penicillin, fourth-generation cephalosporins as well as carbapenems (mainly imipenem). These antimicrobials lower the seizure threshold by competitive antagonism of GABA receptors. If recipients are diagnosed with comorbidities such as renal insufficiency, brain lesions, or pre-existing epilepsy, they are more prone to seizures. With the similar mechanism to β-lactams, fluoroquinolones are another category of proconvulsant antibiotic agents (50, 52, 53). But evidence is only limited to case reports, possibly explained by its lower capability to cross the BBB. As we know, hospitalization medication use of a single patient receiving HSCT is complicated, for this reason, establishing causation between antibiotics and seizures is often confounded by various contributors. Anyway, clinicians should be vigilant when they select antibiotics and define the optimal dosage, if necessary, drug adjustment on an individual basis by closely monitoring of serum levels may help, especially for those predisposed subjects mentioned above. Also, it was reported that foscarnet, an antiviral option in immunocompromised patients, has the risk of causing seizure activity (54). It is noteworthy that drug-drug interaction during the process of HSCT should not be overlooked by prescribers as this phenomenon is related to increasing occurrence of adverse consequences (55, 56). Take the combination of CNIs and azole antifungals for an example, the latter obviously increases the plasma concentration of CsA and the incidence of epileptiform seizures due to potent inhibition of CYP 3A4 (55–57).

### Metabolic Disturbances

For new-onset symptomatic seizures following HSCT, common metabolic disorders (i.e., hyponatremia, hypomagnesemia, hypocalcemia, hypernatremia, and hypoglycemia, etc.) should be excluded and promptly corrected (58), of which the first type usually occurs the most (10, 59). Seizures are more likely to present in cases experiencing severe and rapidly evolving electrolyte imbalances (60), alterations of serum sodium, or osmolality that exerts direct and indirect effects on neuronal discharge mainly by destroying the state of electrical excitability maintained by an ion gradient across cellular membranes. Likewise, patients who suffer from hypomagnesemia and hypocalcemia can develop CNS irritability clinically manifesting with seizures as well (60, 61). As for the pathogenesis of seizures caused by abnormalities in the blood-glucose level, it may be related to neuronal degeneration on account of hypoxia. No matter what kind of disturbance, seizures can be controlled well without sequelae after the underlying reversible factors are properly corrected. In addition, convulsions may be a sole present symptom or one of the performances when CNS involvement secondary to end-stage organ failure after HSCT happens (10, 59, 62). For instance, fulminant hepatitis, liver aGVHD, and hepatic vein occlusion in the context of allografting can result in hepatic encephalopathy and transplantation-related thrombotic microangiopathy/hemolytic-uremic syndrome. CNI nephrotoxicity can cause irreversible kidney injury, and then accumulated toxins in the body cannot be cleared in time, thus resulting in uremic encephalopathy. Whereas such syndromes are mainly dominated by inhibitory neurologic behaviors, the level of consciousness ranges from coma to drowsiness, and seizures are infrequent (62).

### Intracranial Infection

Vascular wall or mucosal damage caused by intensive chemotherapy or total body irradiation, coupled with high doses of immunosuppressive drugs pose a risk for CNS infection in patients undergoing allo-HSCT (63–65). According to changes in their immune status during transplant practice, three phases can be divided: the initial (<30 days after HSCT), intermediate (30–100 days after HSCT), and late (>100 days after HSCT) periods. Intracranial infection can occur at these three distinct time points and predominant microorganisms in each phase differ (64, 66). What is more, it takes time for these immunocompromised patients to reconstitute a functioning immune system, making them more susceptible to opportunistic infection s (67). In contrast to HLA-matched sibling transplantation, patients receiving umbilical cord blood transplantation are more likely to develop a CNS infection (1.7 vs. 8.2%; *P* < 0.001) (68), probably because of a delayed hematologic and immune recovery. The spectrum of major causative pathogenic agents is as follows: fungi such as Aspergillus and Candida, viruses like human herpesvirus-6 (HHV-6), cytomegalovirus, herpes simplex virus, protozoal infection particularly toxoplasma, and G+/G- bacteria (64, 66, 68–71). Convulsions are a common finding in neurologic infections manifesting as meningitis, encephalitis, or brain abscesses, but are of an extremely non-specific nature. In a retrospective analysis of 1,461 cases with allo-HSCT, infections of the CNS were reported in 14 (17.7%) out of 79 patients with seizures (10); the most frequent culprits were fungi and virus, followed by toxoplasma and bacteria. Most CNS viral infections post-HSCT result from a secondary reactivation of a latent pathogen (72), but observations by Santoro et al. (73) indicated it was primary infection rather than reactivation that was associated with subsequent seizures, due to the phenomenon that lower cerebrospinal fluid (CSF) viral load may display a higher likelihood of seizures in pediatric patients infected with HHV6 after transplant; further studies are needed to verify. The above infections can directly damage brain parenchyma and induce seizures (10, 59, 64). In addition, recent studies have demonstrated that inflammatory mediators such as interleukin-1 beta can cause hippocampal damage and imbalance between GABA and glutamate, thus participating in the generation of epilepsy (74, 75). Hence, it is speculated that when post-transplant neurologic infections occur, on one hand, the pathogens may cause local hemorrhage by invading microvessels in the brain, and the accumulation of metabolites can lead to the degeneration or necrosis of neurons, on the other hand, the process of inflammatory cell infiltration and release of various cytokines in the damaged brain regions may also be involved in the epileptogenic effects.

### Cerebrovascular Events

In the post-HSCT setting, the frequency of cerebrovascular diseases including ischemic and hemorrhagic strokes ranges from 2.9 to 4.6% (9, 76, 77), of which intracranial hemorrhage (ICH) is more frequent than brain infractions in some literature (4, 69), while other findings described a similar incidence of hemorrhagic and thrombotic complications (9, 77). Despite a fortunate lower percentage, it is often correlated with dismal prognosis and high mortality. In a retrospective report (*n* = 2,169) of patients undergoing alloSCT, ICH was seen in a total of 32 cases (1.5%), of which 82.9% of the cases were intraparenchymal hemorrhage (IPH), 5.7% of cases were subdural hematoma (SDH), and 8.6% of cases were multiple hemorrhage lesions (78). Another study conducted by Najima et al. (79) found that 21 of 622 (3.4%) allo-HSCT recipients suffered ICH over a 20-year period. The rates of IPH, SDH, and subarachnoid hemorrhage (SAH) were 71.4, 19, and 14.3%, respectively. These phenomena are distinct from previous concepts that SDH appears most often and rarely causes death (69, 70). Most evidence supports the fact that the median time of vascular accidents is around day 100 after transplant (77–80). The common risk factors for developing ICH following allo-HSCT are systemic infections, lower platelet count or fibrinogen levels, and grade III-IV aGVHD, sometimes it is futile even if adequate prophylactic platelets are transfused. In contrast, the occurrence of cerebral thrombotic events may be related to an aberrant coagulative profile or thromboembolism from endocarditis or atrial fibrillation. The clinical picture of cerebrovascular events is usually characterized by headache, convulsions, disturbance of consciousness, paralysis, and so on. It is reported that more than 40% of patients who received HSCT had seizures as part of the clinical presentation of their cerebrovascular diseases (76, 78). The emergence of seizures in the acute stage of cerebrovascular events may originate from a lower threshold for depolarization secondary to substantial glutamate, beyond this, the stimulatory effects of the cortex by blood metabolites and hemosiderin depositions also work. In the later phase, a permanent brain dysfunction covering gliosis, scars, or loss of neurons may form, bringing about abnormal neuronal networks and increased neuronal excitability (81, 82). It is also worth noting that transplantation-associated thrombotic microangiopathy (TA-TMA), a serious complication of allo-HSCT with a mortality rate higher than 70% even after targeted treatment (83, 84), affects multiple systems with widespread endothelial injury and complement activation. Once the CNS is involved, it may manifest as headaches, convulsions, confusion, or hallucinations (84), but there is a paucity of information for the precise incidence of seizures.

### Others

Patients may suffer from various forms of tumors after HSCT, more specifically, the relapse of the CNS, post-transplantation lymphoproliferative disorders (PTLD), and secondary neoplasm. Oshima et al. (85) reported that the cumulative incidence of CNS recurrence after HSCT in leukemia patients was 2.3%, which was one of the important reasons for the failure of transplantation. For PTLD, upon most occasions, it is related to Epstein-Barr virus reactivation and featured by unregulated proliferation of B-lymphocytes. CNS-PTLD can be either disseminated PTLD with CNS involvement or isolated CNS-PTLD, a scarce but life-threatening complication (86). Moreover, patients who received high doses of TBI seem to be particularly liable to develop a new cancer within 10 years or more, such as meningioma, glioblastoma, and astrocytoma, etc. The clinical symptoms of sufferers with all these three conditions are non-specific and consist of seizures, altered mental status, or focal neurological deficits. Given their rarity in clinic, few controlled studies have evaluated the rate and time course of a certain manifestation like convulsions. Last but not the least, neurological presentations of chronic GVHD such as vasculitis and immune-mediated encephalitis, albeit less likely, are another element causing epileptic seizures (87, 88). Their pathological hallmarks are the infiltration of donor-derived monocytes and CD3+CD8+ cytotoxic T cells into the brain parenchyma (89), a striking histological homophyly to pathological specimens after surgical resection in patients with temporal lobe epilepsy (90). This finding may provide a clue for some unexplained posttransplant seizures, and of course, further investigation is required.

## Clinical Features

In terms of the onset timing of transplantation-related seizures, Zhang et al. (10) have reported that up to 90% of seizures occur within 1 year after HSCT, especially in the first 100 days, which accounted for more than three-fifths of all transplanted patients with seizures. Only a limited number of patients (3.8%) had convulsions at the conditioning phase, while seizures caused by CNS relapse generally appeared after the first year. To be more specific, seizures induced by Bu toxicities typically happen between the second day of Bu administration and 24 h following the final dose of Bu owing to its accumulation (16). PRES after HSCT, a clinical neuroradiologic entity with an incidence of 1.1–8.3% (6, 91–93), preferentially presents within the first month (6, 42, 94, 95). Zhang et al. (10) also found that the median interval of seizures is 56 days (range of −6 to +880), which is similar to a smaller-sized study conducted for children by Cordelli et al. (12), who documented a median period of 78 days after infusion of stem cells (range −3; +352). From the perspective of the overall frequency, 16 out of 28 transplanted patients with seizures have a single course, and repeated seizure often happen within 7 days after onset (12), only a few cases finally developed refractory epilepsy, which were mostly related to taking CsA orally (96).

After searching the related literature about seizure types, it can be concluded that both generalized and focal seizures have been reported in patients receiving HSCT, and the former is in the majority, especially generalized tonic-clonic seizures (GTCS), but prospective research on this topic is still scarce. Zhang et al. (10) analyzed recipients of allo-HSCT between 2004 and 2010 at their institute and summarized that those who experienced generalized-onset seizures occupied about 65% of all sorts of attacks, followed by complex partial seizures and simple partial seizures. Some other seizures like partial seizures progressing into grand mal or absence seizures have been documented as well (59). Dating back to etiologies, as most generalized seizures are associated with CNI administration (27, 97), they may be accompanied by some mental and behavioral abnormalities or just occur in isolation, and seizures can be terminated after reducing the dosage, drug discontinuation, or switching to other immunosuppressive drugs. In addition, GTCS is also one of the most common seizure types caused by Bu. When it comes to post-transplanted PRES, occipital lobe seizures and status epilepticus (SE) are the common seizure types described in this syndrome, in particular, SE is most frequently observed in children (91, 98). The initial symptoms of the first seizure may only be a tiny twitch or some focal signs of non-convulsive manifestation, such as gaze deviation, eye clonus, visual hallucinations, or just changes in mental state. An electroencephalogram (EEG) examination at this time is necessary to confirm diagnosis followed by intervention without delay. Focal seizures are common in patients with local structural alteration in the brain, such as intracranial hemorrhage, encephalitis, and brain abscess, etc. The abnormal discharge of neurons originates from one side of the cerebral hemisphere, which can be manifested as motor and sensory syndromes or simply a minor decreased level of consciousness, some of them probably evolve into generalized convulsions. Univariate and multivariate statistical analysis pointed out that the age of recipient (≤18 years), haploidentical transplantation, and aGVHDs are high risk factors for seizures, probably explained by incomplete development of the nervous system in children and the increased dosage of immunosuppressants to prevent GVHD, thereby increasing the vulnerability to infection and drug toxicity (10). However, this result differs from that identified in pediatric HSCT (12), concluding that cord blood stem cell (CBSC) transplantation and non-oncological diseases were the influencing factors for seizures. Diverse transplant procedures and population characteristics of different age groups may give a possible explanation for this phenomenon.

After the attack of the first seizure following allo-HSCT, doctors are advised to expeditiously conduct blood glucose, electrolyte, blood routine examination, inspections of liver, kidney functions, and immunosuppressant concentration measurements to determine the underlying causes, and equally important, prompt adjustment of treatment plans to prevent irreversible damage to the brain. If CNS infection or recurrence is suspected, a lumbar puncture and culture of blood and urine are needed to seek for ancillary evidence. The changes in cerebrospinal fluid during the early stage of cranial infection may be inconspicuous, so a repetitive submittal for inspection is necessary as the disease progresses to find out the relevant pathogen or infiltrated tumor cells. Moreover, imaging examination (computed tomography and magnetic resonance imaging) also provide certain vital references for judging the cause and severity of seizures after HSCT (66, 86). In most cases, a CT scan is the first-line modality despite of a lower sensitivity and detectable rates than MRI, mainly employed to exclude hemorrhagic diseases. For example, a large patch of high signal intensity on T2-weighted and FLAIR is particularly suggestive of CsA neurotoxicity, and the lesions are usually bilateral, symmetrical, and located predominantly in the posterior cerebral white matter, and sometimes is also involved the cortex (29, 98, 99). If the limbic system including temporal lobe, hippocampus, and amygdala has hyperintense lesions, it may resemble the typical imaging appearances of HHV-6 infection (66, 73, 100). Another diagnostic tool is EEG, which can assist with the determination of seizure type and therapeutic guidance, especially when patients are in a coma. It is of great significance to discern non-convulsive status epilepticus (58, 97). For patients suffering from PRES, whose EEG abnormalities behave like periodic lateralized epileptiform discharges or spike discharges deriving from parieto-occipital or occipital-temporal regions precede neuroradiological changes, an urgent EEG test can aid in earlier diagnosis and management (12, 58, 91).

## Therapy and Prognosis

### Etiological Treatment

In the early stage of convulsions, it is key to accurately decide the causes and eliminate them in time, such as correcting electrolyte derangements, regulating blood glucose, and controlling blood pressure, emergency platelet transfusion, and anti-infection, etc. If a seizure is considered to be induced by CNIs, after weighing long-term efficacy against the risk of rejection, replacing the medication or lowering its dosage may help with the termination of attack. Other generic drugs can be combined or replaced to avoid irreversible brain damage caused by non-immediate disposal. There were also other promising therapeutic strategies put forward aiming at improving immunosuppressant-induced seizures after organ transplantation (101, 102), like lipid supplementation and combinations of CsA with N-(30,40-dimethoxycinnamonyl) anthranilic acid (3,4-DAA), an agent known to suppress immune response through inducing the apoptosis of activated T cells, but there have been no such reports in CNIs-induced seizures after allo-HSCT. In general, most of these seizures do not require long-term treatment with anti-epileptic drugs (AEDs) if only removing reversible incentives. A close observation of signs alone in clinic is required.

### Pharmacological Therapy

The overall goals of treating post-transplanted patients with seizures have been published in a review by Shepard et al. (58), which are in accordance with those who suffer from epilepsy. Besides recognizing and dislodging underlying causes, controlling seizures with AEDs is of great significance. When SE occurs after allo-HSCT, intravenous use of benzodiazepines or midazolam is deemed first-line therapy, if it is not stopped and an intractable SE develops, anesthetics (such as propofol) with anticonvulsive effects can be administrated under the circumstances of essential life support (12, 99). Nevertheless, prognosis of these patients is unfavorable and mortality is high. For sufferers of acute repetitive seizures or a single epileptic episode along with potential recurrence risk on EEG and/or cerebral imaging, or even when the underlying epileptogenic cause is uncertain, oral AEDs like levetiracetam or gabapentin should be initiated according to the suspected seizure type (52, 58, 103), most patients can benefit a lot from them. For example, Cordelli et al. (12) and Zhong et al. (59), respectively, reported that 96.43 and 75% of recipients had seizure termination after taking anticonvulsants. The total duration of drug therapy is 1–3 months in general (104), a longer time should be ensured for those transplanted candidates with organic lesions. In any case, the principle of selecting appropriate AEDs is trying to avoid those with strong drug interactions, liver enzyme-inducing properties, and potent inhibitory effects to hematopoietic functions. Unfortunately, there is still no definitive guidelines or expert consensus to refer to for such a patient group, so the final therapeutic regimen mostly relies on experience in clinical practice. As mentioned above, levetiracetam is the first choice to semeiologically treat concurrent seizures after transplantation because of its favorable features. Surprisingly, Peyrl et al. (105) reported a case of secondary graft failure after HSCT because of treatment-related myelodysplastic syndrome during the period of levetiracetam administration, and believed that levetiracetam was the possible cause. Therefore, studies on the safety profile of levetiracetam remain to be evaluated further in post-transplanted patients with seizure attacks.

### Treatment Outcomes

A retrospective study conducted by Cordelli et al. (12) found that the 5-year overall survival for patients with seizures was statistically lower when compared to those without seizures, which is consistent with the conclusion drawn by Zhang et al. (10). After a follow-up of 1,461 patients with HSCT for 6 years, the transplantation-related cumulative survival rate of recipients experiencing seizures declined dramatically, with 71.4 and 31.1%, respectively. Meanwhile, 42 of 79 patients with epileptiform seizures died, of which 17 patients succumbed to uncontrolled seizures or related causes. Subgroup analysis showed that different reasons for epileptic seizures after HSCT could exert different impacts on survival conditions. For example, the mortality of patients undergoing epileptic seizures caused by intracranial infection and cerebrovascular disease was relatively high and the prognosis was dismal, while PRES seldom had effects on survival, often ending with a good outcome. Another phenomenon worth noting is that patients who showed postoperative SE had a high death rate and great attention should be paid to it in clinic.

## Conclusions and Perspectives

Epileptic seizures in patients of allo-HSCT are not infrequent in clinical practice, increasing attention has been paid to it by clinicians due to its poor prognosis. However, research on this issue is still limited up to now. Most studies are retrospective, with some authors including patients with a previous history of epilepsy or existing brain damage when gathering data. Therefore, a further expansion of sample size and targeted prospective studies should be attempted in the future. At the present stage, etiologies of epileptic seizures after transplantation mainly focus on drug-related neurotoxicity, metabolic disorder, cerebrovascular events, intracranial infection, and CNS recurrence. However, sometimes seizures may be the result of the co-existence of multiple possible reasons, for instance, cerebral infection itself can simultaneously lead to brain damage and hemorrhage by invading blood vessels, and some antibiotics utilized to combat infection may also induce seizures. Based on this fact, investigations into the interaction between varieties of factors can be strengthened. In addition, a small number of patients with unknown etiology might be related to immune encephalitis caused by cGVHD, deeper studies about immune mechanism seem to be promising. Management and prognosis is determined by whether seizures are systemic and usually reversible, such as derangement (e.g., hyponatremia, drug toxicity) or a structural CNS disorder. In summary, clinicians should enhance the understanding of convulsions during the process of HSCT. A close multidisciplinary collaboration between hematologists, neurologists, and radiologists is significant to achieve early diagnosis and timely treatment, thereby ensuring optimal care of such sufferers, reducing mortality, and improving their clinical outcomes.

## Author Contributions

All authors contributed to the design, writing, critical revision of this review, and approved the final version of the manuscript.

## Conflict of Interest

The authors declare that the research was conducted in the absence of any commercial or financial relationships that could be construed as a potential conflict of interest.
